# Chronic Neuropsychological Sequelae of Cholinesterase Inhibitors in the Absence of Structural Brain Damage: Two Cases of Acute Poisoning

**DOI:** 10.1289/ehp.7545

**Published:** 2005-02-10

**Authors:** Lola Roldán-Tapia, Antonia Leyva, Francisco Laynez, Fernando Sánchez Santed

**Affiliations:** ^1^Departamento de Neurociencia y Ciencias de la Salud, Universidad de Almería, Almería, Spain;; ^2^Hospital de Poniente, Almería, Spain

**Keywords:** central nervous system dysfunction, long-term sequelae, neuropsychological profile, pesticide poisoning

## Abstract

Here we describe two cases of carbamate poisoning. Patients AMF and PVM were accidentally poisoned by cholinesterase inhibitors. The medical diagnosis in both cases was overcholinergic syndrome, as demonstrated by exposure to cholinesterase inhibitors. The widespread use of cholinesterase inhibitors, especially as pesticides, produces a great number of human poisoning events annually. The main known neurotoxic effect of these substances is cholinesterase inhibition, which causes cholinergic overstimulation. Once AMF and PVM had recovered from acute intoxication, they were subjected to extensive neuropsychological evaluation 3 and 12 months after the poisoning event. These assessments point to a cognitive deficit in attention, memory, perceptual, and motor domains 3 months after intoxication. One year later these sequelae remained, even though the brain magnetic resonance imaging (MRI) and computed tomography (CT) scans were interpreted as being within normal limits. We present these cases as examples of neuropsychological profiles of long-term sequelae related to acute poisoning by cholinesterase inhibitor pesticides and show the usefulness of neuropsychological assessment in detecting central nervous system dysfunction in the absence of biochemical or structural markers.

Poisoning events and chronic exposure to cholinesterase inhibitors, organophosphates (OPs), and carbamates have traditionally been associated with neurotoxic consequences, such as poor neurobehavioral performance in some cognitive domains such as information processing and memory (Abou-Donia 2003; Wesseling et al. 2002; Yokoyama et al. 1998) or delayed neuropathy induced by certain OPs (Eyer 1995; Jamal 1997). The following two cases show neurocognitive deficits after different types of poisoning events by cholinesterase inhibitors (carbamates and OPs): the first case (AMF) was due to accidental ingestion of a carbamate compound, and the second (PVM) was a greenhouse worker with a history of repeated poisonings while working with OPs, carbamates, or both together. Both patients received emergency-room care for overcholinergic syndrome by cholinesterase inhibitors. Three and 12 months later, cholinesterase levels and neuropsychological performance were assessed following protocols proposed for neurotoxicology evaluation (Fiedler 1996; White and Proctor 1995).

## Case 1

**Figure 1:**
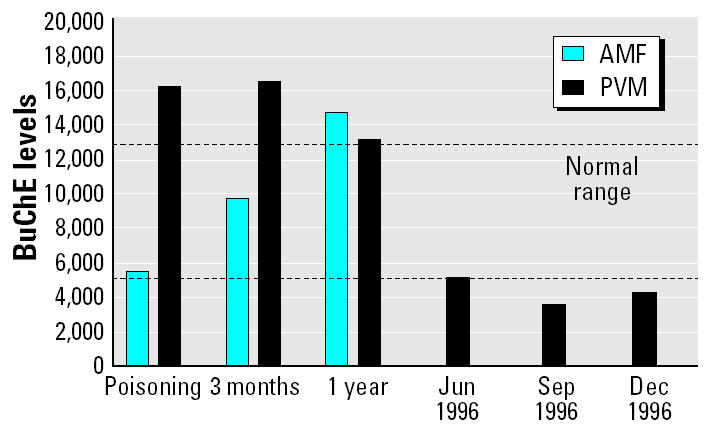
Plasmatic cholinesterase levels (BuChE) of both patients immediately after poisoning and at 3 months and 1 year later, as well as enzymatic activity levels of PVM’s previous intoxications in 1996. The two dashed lines indicate the normal range.

In January 1998, AMF, a 55-year-old right-handed female, was attended at the emergency room in the Hospital de Poniente (Almería, Spain) after accidentally drinking a glass of methomyl, a carbamate pesticide. This compound had been prepared by her son and kept in the refrigerator in a bottle for later use in the greenhouse. AMF drank a glass, thinking it was a refreshment. Half an hour later, she was taken to the hospital by her husband, suffering from physical discomfort. Upon the arrival at the emergency room, she presented a Glasgow Coma Scale (GCS) of 15, and her symptoms included perspiration, tremors, myosis, respiratory problems, sialorrhea, and vomiting. The patient did not present hypoxia, coma, or loss of consciousness [butyrylcholinesterase (BuChE) levels shown in Figure 1]. She was treated in the hospital with gastric lavage, activated charcoal, cathartics, and antiemetics. She was released 1 week later without further medical treatment. No neurological or physical disturbances were observed. Once at home, she reported slowness, subtle disorientation, and attention and memory problems in daily activities, such as recalling telephone numbers or cooking.

A year later in a medical follow-up visit, she reported that she felt well: Orientation and speed had improved, as had competency in routine abilities such as cooking, but memory problems remained. No other physical problems were recorded. The brain computed tomography (CT) done at this time was considered normal.

### Summary of neuropsychological functioning.

AMF underwent a broad-based clinical neuropsychological examination in April 1998 and April 1999. For some of the tests [Logical Memory test and Rey Auditory Verbal Learning tests (Rey 1964)], an alternative form was used in the second evaluation, to avoid effects of learning and practice. During the interview, AMF reported a low level of education, not having finished obligatory primary school. Her primary role was as a housewife, managing money and the home, raising her children, and sometimes helping in agricultural work. Her estimated IQ was 95 (Bilbao-Bilbao and Seisdedos 2004), and her corrected score on the Mini Mental State Examination (Lobo et al. 1979) was 28, indicating the absence of dementia. She adequately performed tests of single-word reading, basic written arithmetic, and semantic knowledge. Her writing skills were not evaluated, except for her name. Each outcome was evaluated in relation to her educational level, sex, estimated IQ, and age, bearing in mind that the variable “education” has an influence on an individual’s overall neuropsychological performance.

In the first assessment, her performance was below expectation for her estimated pre-morbid IQ in the domains of attention, memory, motor skill, and constructional abilities (Table 1). A minimal depression was also registered.

## Case 2

PVM, a 26-year-old right-handed male, was most recently poisoned (February 1998) by a mixture of pesticides [carbamate (methomyl) and pyrethroid (cypermethrin)] while spraying in a greenhouse without any personal safety equipment. PVM attended the emergency room at the Hospital de Poniente (Almería, Spain) because of cephalalgia, abdominal pain, and vomiting. His GCS was 15 (BuChE levels shown in Figure 1). He was given a 2-week prescription for atropine (1.2 mg) and antiemetics and was released the next day. He had been poisoned with OPs and carbamates six times previously (all due to the absence of personal safety equipment), with three of them documented. The first took place in June 1996 while he was spraying with methomyl; upon arrival at the hospital, his symptoms were dizziness and perspiration. The second happened in September 1996 while he was working with a mixture of methomyl and chlorpyrifos (OP); in addition to cholinesterase inhibition, the patient showed tremors, perspiration, respiratory problems, sialorrhea, and vomiting. The third documented poisoning event happened in December 1996 when he was spraying with a mixture of methomyl and chlorpyrifos; his symptoms were vomiting, myosis, abdominal pain, perspiration, and respiratory problems. In these four events, the medical diagnosis was overcholinergic syndrome. No coma, hypoxia, or convulsions were recorded in any of these events. In all cases, treatment in the hospital was gastric lavage and atropine, and the poisonings were resolved in < 24 hr.

PVM underwent cognitive testing in May 1998 and May 1999. During the first interview, he reported mnesic problems, such as remembering telephone numbers and the events of the previous day, but complained of no physical symptoms. He had completed secondary school and had been working in agriculture for 10 years. His estimated IQ was 105, and he obtained a score of 30 in the Mini Mental State Examination. Each outcome was evaluated in relation to his educational level, estimated IQ, sex, and age. Tests of single-word reading, writing skills, basic mental, and written arithmetic and semantic knowledge were completed correctly. Physical and neurological assessment did not show any alteration at the time of the neuropsychological testing. He did not receive pharmacological treatment.

During the second assessment in May 1999, he reported that he continued working in the greenhouse. No further poisoning events had been recorded during this time. The magnetic resonance image (MRI) taken a year after the last poisoning event did not reveal any evidence of brain injury. He reported no physical complaints.

### Summary of neuropsychological functioning.

In 1998, PVM’s performance was below expectation for estimated premorbid abilities in the domains of motor skills and short and long-term memory. Deficits at the level of learning new information were detected on several tasks, but he showed forgetting of information over a delay only in visuospatial tasks. His outcomes reflected slowness in the copying of a complex figure or the fulfillment of a complex attentional task (Table 1).

When comparing his 1999 performance with that of 1998, the speed of processing appeared to improve, although probably due to a practice effect. Yet, disturbances remained in the short- and long-term logical memory, as well as in visual memory, and in motor tasks such as alternation and coordination, which implies programming and motor regulation injury. His score for depression was in the normal range, whereas the Taylor Anxiety Scale score (Taylor 1953) showed a subtle increase, although still in normal range. During the assessment sessions, the patient was cooperative but very worried about the possibility of sequelae.

## Discussion

Cholinesterase inhibitors, OPs, and carbamates are powerful insecticides widely used in agriculture. However, they are acutely toxic to humans and may cause poisoning as a result of exposure in the workplace, or as accidental or suicidal events (Fengsheng 2000). This is the case in the intensive agriculture industry in southern Spain, where a large number of intoxications have been documented. In only the first half of 2000, 49 occupational poisoning events were recorded [Sociedad Española de Sanidad Ambiental y Asociación Española de Toxicología (SESA/AET) 2000]. The toxic compounds that produced those events were insecticides, mainly OPs (59%), followed by carbamates (34%) and organochlorides (10%).

The main neurotoxic reaction after absorption of cholinesterase inhibitors is acute cholinergic syndrome due to the inhibition of the acetylcholinesterase (AChE) enzyme, which is reversible in case of the carbamates and irreversible in case of OPs. This inhibition leads to an accumulation of acetylcholine (ACh) at synapses, causing overstimulation and subsequent disruption of transmission of impulses in the central, peripheral, and autonomic nervous systems (Martín-Rubí et al. 1995; Storm et al. 2000). Symptoms in patients who experience cholinesterase inhibitor poisonings may include dry mouth, fasciculation, tremor, agitation, ataxia, weakness (Steenland 1996), tension, anxiety, irritability, restlessness, and headaches (Stephens et al. 1995). Once the cholinergic imbalance has been corrected, many of the symptoms usually disappear.

Several studies have shown the existence of both short- and long-term neuropsychological symptoms after acute intoxication by pesticides, mainly OPs. The first publications of acute effects reported neurocognitive sequelae, anxiety, irritability, insomnia (Tabershaw and Cooper 1966), loss of memory (Holmes and Gaon 1956), and reactions similar to schizophrenia and depressive symptoms (Gershon and Shaw 1961). The first controlled study assessing workers who suffered acute poisoning from cholinesterase inhibitor compounds and/or organochlorides was reported by Savage et al. (1988). The poisoned group showed deteriorated intellectual functioning, academic skills, abstraction, reasoning, motor skills, and sensitivity to social stress. No significant difference between poisoned subjects and controls was found on audiometric tests, ophthalmic tests, electroencephalograms, or clinical serum and blood biochemistry evaluations (Savage et al. 1988). Later, Rosenstock et al. (1991) reported a retrospective study in which 36 workers were tested, all of whom had suffered acute OP intoxication 1–3 years earlier. Lifetime work experience data (years worked, other toxics) and recent exposure were controlled. Rosenstock et al. (1991) found evidence of brain damage with impairment of short-term memory, attention, sequencing and problem solving, visuospatial cognition, and depression. A third study compared an OP-poisoned group with control subjects matched in sex, age, and educational level (Steenland et al. 1996). Cholinesterase inhibition was also registered for the poisoned group. Tests included a neurological physical examination, and nerve conduction, vibrotactile sensitivity, neurobehavioral, and postural sway tests. The results pointed to neurocognitive deficits and disturbed peripheral nerve function. The poisoned group had poor scores in neuropsychological tests, such as sustained attention, and showed confusion and tension in the mood scales (Steenland et al. 1996).

In another study, Yokoyama et al. (1998) assessed neuropsychological sequelae in a small group of people who had suffered acute sarin poisoning in a terrorist attack 6–8 months earlier. The authors assessed serum cholinesterase activity in patients on the day of poisoning, controlling for age, education level, alcohol consumption, and smoking status. Deficits in psychomotor performance and posttraumatic disorders, together with disturbances in brain-evoked potentials, were found.

In a recent study carried out in Costa Rica, Wesseling et al. (2002) compared neuro-behavioral performance between two groups of farmers with previous acute intoxications by OP or carbamates. Plasma cholinesterase was assessed for each group of subjects. Two years later, the patients showed long-term sequelae: deficits in visuo- and psychomotor tasks. Performance of the OP-poisoned subjects was worse than that of the farmers who had been poisoned by carbamates.

Although the dysfunctions found are different depending on the task, the type of pesticide, and the severity of poisoning, the three studies (Rosenstock et al. 1991; Wesseling et al. 2002; Yokoyama et al. 1998) have a common profile of deteriorated intellectual functioning, academic abilities, distress, motor skills, posttraumatic stress, confusion, and tension, as well as self-reported symptoms of depression, irritability, and confusion.

The pattern of cognitive deficit that we report here is generally quite typical of the pattern of deficits reported after acute poisoning events. AMF and PVM displayed decreases in the speed of processing, visuospatial functioning, and short-term visual and logical memory deficits. Programming motor activity was also damaged, and minor anxiety was recorded. In both cases, neuropsychological dysfunction remained a year later in the absence of biochemical abnormality or structural brain damage, as shown by CT scan and MRI, in line with several previous reports (Jamal 1997).

It could be argued that the results obtained by AMF on the Beck Depression Inventory (Beck et al. 1961) may explain some of the deficits found. Some authors (Buckelew and Hannay 1986; Gass and Daniel 1990; Meyers and Meyers 1996) suggest that high degrees of depression or anxiety can produce alterations in neuropsychological performance (including some of the tests we used, e.g., Rey-Osterrieth Complex Figure (ROCF), Trail-Making Test B, block design; Table 1). AMF suffered a minimal degree of depression that had mostly disappeared a year later, whereas neurobehavioral deficits remained; thus, it seems that affective deregulation cannot account for our results for AMF.

Repeated administrations of the same test can produce practice effects, especially in tests of verbal memory (Benedict and Zgaljardic 1998; Theisen et al. 1998). To avoid this learning effect, alternative forms of Logical Memory and Rey Auditory Verbal Learning (RAVL) tests were used for retesting both patients. Nevertheless, very small increases occurred in the second administration of the nonverbal tests, such as the score on the time to copy and the recall of the complex figure. In general, the subtle improvement shown in the second assessment indicates a recovery effect, which reinforces the idea that neither education nor poor intellectual abilities fully explain the deficits in neuropsychological performance.

Finally, the last poisoning event suffered by PVM was due to a mixture of carbamate and pyrethroid. Although pyrethroid has been considered among the safest classes of insecticides available (Dorman and Beasley 1991), there have been few reports of systemic poisoning in humans by pyrethroid insecticides. In a review on this topic, Müller-Mohnssen (1999) reported that the symptoms after an acute intoxication (burning sensation of eyes and face, painful irritation of respiratory mucosa, vertigo, and disturbed consciousness) and the period of latency and symptoms of subacute intoxication (tingling, sensation of burning, and sensibility disorder) are dissimilar to those reported after OP and/or carbamate poisoning events, and dissimilar to those presented by PVM upon arrival at the hospital. In fact, it is difficult to attribute the neuropsychological deficit to pyrethroid intoxication when PVM’s diagnosis was overcholinergic syndrome by cholinesterase inhibitors, corroborated by the symptomology presented, which was similar to that previously recorded after intoxications with cholinesterase inhibitors, and the certainty of contact with the carbamate methomyl.

The pharmacological changes in the cholinergic system could be related to the cognitive deficits found. The mechanism of this neurotoxic effect is uncertain. Bushnell et al. (1995) demonstrated a phenomenon of tolerance to repeated doses of chlorpyrifos (OPs) in rats due to the synaptic adaptation of muscarinic receptors (down-regulation). However, this adaptation has a functional cost: The rats showed cognitive deficits, even when the cholinesterase level had returned to normal.

The inhibition of brain AChE by carbamates affects different subtypes of neuronal nicotinic receptors, independently of AChE inhibition. This implies that neuronal nicotinic receptors are additional targets for some carbamate pesticides and that these receptors may contribute to carbamate pesticide toxicology, especially after long-term exposure (Smulders et al. 2003).

Perhaps the underlying mechanism should be sought in the role of the cholinesterase enzyme. A current hypothesis proposes a compensatory mechanism with functional consequences, whereby cholinesterase activity increases after the poisoning events, which is explained by cholinesterase being quickly synthesized in response to brain hyperexcitability after cholinesterase inhibitor poisoning events and after stress (Meshorer et al. 2002). AChE inhibition causes an increase in ACh, activating pre- and postsynaptic cholinergic receptors. There is an immediate transcriptional regulation of genes coding for AChE, choline acetyltransferase (ChAT), and vesicle ACh transporter (VAChT), which reduces the expression of ChAT and VAChT mRNA, increasing the AChE mRNA (Grisaru et al. 1999).

This may be the case for PVM, who after six poisoning events had very high levels of plasma cholinesterase. Indeed, a year later, both AMF and PVM showed above-normal BuChE (Figure 1). BuChE, also called pseudo-cholinesterase or plasma cholinesterase, is an enzyme genetically different from AChE, although they share some important functions, such as ACh hydrolysis (Darvesh et al. 2003). Individual susceptibility to cholinesterase inhibitor compounds is due, in part, to individual genetic variations of this enzyme (Fontoura-da-Silva et al. 1996). Nevertheless, it is widely used as a biomarker of both exposure to cholinesterase inhibitors and recovery from acute intoxications. A cholinergic crisis, together with reduced levels of plasma BuChE activity, leads to the diagnosis of overcholinergic syndrome (Martín-Rubí et al. 1995). Research in progress by our group points to a strong correlation between the number of poisoning events and plasmatic cholinesterase level, which is higher in subjects who have undergone several poisoning events (Roldán-Tapia et al. 2000).

The underlying mechanism might be found in the noncatalytic functions of cholinesterases. Current studies support the idea of a trophic function of the G1 form, and possibly the G4 form, of this enzyme in the central nervous system and neuromuscular junction during development (Andres et al. 1997, 1998; Darvesh et al. 2003). The toxic effect of the synthesis of different forms of the enzyme is unknown but might explain functional damage in the central nervous system. In this regard, it has been shown that an overexpression of AChE in transgenic mice produces progressive neurochemical, neuromorphological, and neurocognitive alteration, at least in spatial memory in adult mice (Andres et al. 1997, 1998; Beeri et al. 1997, 1998). These studies lead us to believe that changes in enzymatic level may produce a pathological dysfunction that explains the neuropsychological deficits found after poisoning by cholinesterase inhibitors such as OP compounds and carbamates (Kaufer et al. 1998).

## Conclusions

Pesticide poisoning is a serious health problem that affects the general population, specifically people working with these compounds. Pesticides are designed to kill, reduce, or repel insects, fungi, and other organisms that can threaten public health. However, when improperly used or stored, these chemical agents can also harm humans. Key risks are cancer, birth defects, and damage to the nervous system and to the functioning of the endocrine system. Pesticides are known to cause millions of acute poisoning cases per year, of which at least 1 million require hospitalization. Between one and three agricultural workers per every 100 worldwide suffer from acute pesticide poisoning [United Nations Environment Programme (UNEP) 2004]. The contribution of pesticides to chronic diseases, on the other hand, is unknown. Tackling the risks of pesticide exposure and poisoning requires comprehensive strategies. These strategies should be designed at the local level and supported regionally, nationally, and internationally. They should include research activities on how to develop effective economic and legal instruments. In addition, they should ensure that the public is informed, that health conditions are monitored and, where necessary, that treatment programs are established.

At the clinical level, our findings, together with previous research, provide evidence that cholinesterase inhibitor poisoning has both short- and long-term neuropsychological sequelae, through cholinesterase inhibition and/or other unknown mechanisms, demonstrating the utility of neuropsychological examinations in detecting secondary central nervous system dysfunction after pesticide intoxication. Follow-up studies, controlling for the type and amount of pesticides, AChE level, different forms of the enzyme, and more specific neurobehavioral tasks, will be needed to demonstrate a possible effect of these substances on the central nervous system.

## Figures and Tables

**Table 1:** Test scores of patients AMF and PVM on tests of general cognitive abilities and emotional state, and interpretation according to accepted cutoff points.

	AMF			PVM		
Test	3 months	1 year	Cutoff norms	3 months	1 year	Cutoff norms
Attention						
Digit span WAIS backward*^a^*	3	3	4.1 ± 0.6^b,c^	4	5	4.30 ± 1.11
Stroop test	NA	NA		52	52	< 50th percentile*^d^*
Letter cancellation A	3 errors	2 errors	0 errors*^e^*	0 errors	0 errors	0 errors*^e^*
Trail-Making A	NA	NA		40 sec	42 sec	Test 18.5 ± 5.1*^c^* Retest 16.2 ± 5.0
Trail-Making B	NA	NA		90 sec	92 sec	Test 41.6 ± 11.4*^c^* Retest 34.0 ± 12.7
Digit symbol WAIS	5	7	> 10	12	12	> 10
Reasoning						
Picture completion WAIS	7	8	> 10	12	12	> 10
Similarities WAIS	4	6	> 10	14	12	> 10
Memory						
Digit span WAIS forward	4	4	4.9 ± 0.8*^c^*	5	5	5.98 ± 1.12*^c^*
RAVL test (trial 1–5)*^f^*	2, 5, 6, 8, 8	3, 5, 7, 8, 8	Means 4.86, 6.81 8.66, 9.40, 9.71*^c^*	4, 6, 10, 11, 13	4, 6, 9, 12, 13	Means 7.4, 10.5, 12.2, 13.0, 13.4*^c^*
RAVL test after delay	5	6	7.81 ± 3.7	11	11	12.1 ± 2.8
Logical Memory A, immediate recall (WMS)*^g^* 5		5	13.0 ± 2.3*^h^*	10	7	13.8 ± 6.0*^h^*
Logcal Memory A after delay	5	4	10.2 ± 4.0	8	6	13.0 ± 5.0
ROCF immediate recall	9	10	14.45 ± 5.3*^e^*	11.5	12	18.88 ± 6.1*^e^*
ROCF delayed recall	9.5	10	13.4 ± 6.0*^e^*	12	12.5	20.00 ± 6.4*^e^*
BVRT*^i^*	9	11	12.8	13	12	13.7
Visuospatial/visual motor						
ROCF copy quality	10	10	30.5 ± 4.7*^c^*	25	28	33.0 ± 2.8*^c^*
ROCF copy time	5 min 20 sec	4 min 30 sec	< 3 min*^j^*	3 min 56 sec	3 min 50 sec	> 3 min*^j^*
BVRFT*^h^*	26	27	Mean 31.00*^k^*	28	28	Mean 31.00*^k^*
Astereognosis	5	5	Mean 5*^e^*	5	5	Mean 5*^e^*
Poppelreuter’s test	10	10	9 ± 1*^e^*	10	10	9 ± 1*^e^*
Block design WAIS	5	6	> 10	12	12	> 10
Ideomotor praxis	0 errors	0 errors	0 errors*^l^*	0 errors	0 errors	0 errors*^l^*
Ideational praxis	0 errors	0 errors	0 errors*^l^*	0 errors	0 errors	0 errors*^l^*
Reciprocal inhibition	0 errors	0 errors	0 errors*^e^*	3 errors	1 error	0 mistakes*^e^*
Motor alternate	Not*^m^*	Not	Correct*^e^*	Not	Not	Correct*^e^*
Motor coordination	Not*^n^*	Not	Correct	Correct	Correct	Correct
Rhythm reproduction	4	6	9 ± 2*^e^*	4	5	10 ± 2*^e^*
Language						
Boston Naming Test*^o^*	45	43	49.2 ± 5.6*^c^*	54	55	57.8 ± 2.1*^c^*
Emotional status						
Beck Depression Inventory	14	10	10–15: minimal depression	6	6	0–9 normal range
Taylor Anxiety Scale*^p^*	33	21	16–45	18	20	14–45

Abbreviations: BVRFT, Benton Visual Recognition Form Test; BVRT, Benton Visual Retention Test; NA, not available; RAVL, Rey Auditory Verbal Learning; ROCF, Rey-Osterrieth Complex Figure; WAIS, Wechsler Adult Intelligence Scale; WMS, Wechsler Memory Scale.

a Weschler (1993).

b Mean ± SD.

c Mitrushina et al. (1999).

d Golden (1994).

e Lezak (1995).

f Rey (1964).

g Weschler (1997).

h Spreen and Strauss (1998).

i Benton et al. (1994).

j Rey (1997).

k Rey and Sivan (1995).

l Strub and Black (1988).

m The same sequence of alternation in two essays of seven trials each.

n The movement was not correctly performed in either of the trials.

o Goodglass and Kaplan (1990).

p Taylor (1953).
